# Computer-assisted automatic synthesis II. Development of a fully automated apparatus for preparing substituted N–(carboxyalkyl)amino acids

**DOI:** 10.1155/S1463924689000428

**Published:** 1989

**Authors:** Nobuyoshi Hayashi, Tohru Sugawara, Motoaki Shintani, Shinji Kato

**Affiliations:** Central Research Division Takeda Chemical Industries Ltd. Juso Honmachi 2-chome Yodogawa-ku Osaka 532 Japan

## Abstract

A versatile automated apparatus, equipped with an artificial intelligence has been developed which may be used to prepare and isolate a wide variety of compounds. The prediction of the optimum reaction conditions and the reaction control in real time, are accomplished using novel kinetic equations and substituent effects in an artificial intelligence software which has already reported [1]. This paper deals with the design and construction of the fully automated system, and its application to the synthesis of a substituted N-(carboxyalkyl)amino acid. The apparatus is composed of units for perfoming various tasks, e.g. reagent supply, reaction, purification and separation, each linked to a control system. All synthetic processes including washing and drying of the apparatus after each synthetic run were automatically performed from the mixing of the reactants to the isolation of the products as powders with purities of greater than 98%. The automated apparatus has been able to run for 24 hours per day, and the average rate of synthesis of substituted N-(carboxyalkyl)amino acids has been three compounds daily. The apparatus is extremely valuable for synthesizing many derivatives of one particular compound structure. Even if the chemical yields are low under the optimum conditions, it is still possible to obtain a sufficient amount of the desired product by repetition of the reaction. Moreover it was possible to greatly reduce the manual involvement of the many syntheses which are a necessary part of pharmaceutical research.


Journal of Automatic Chemistry Vol. 11, No. 5 (September–October 1989), pp. 212–220

### Computer-assisted automatic synthesis II. Development of a fully automated apparatus for preparing substituted N-(carboxyalkyl)amino acids

**Nobuyoshi Hayashi, Tohru Sugawara, Motoaki Shintani and Shinji Kato**

*Central Research Division, Takeda Chemical Industries, Ltd. Juso Honmachi 2-chome, Yodogawa-ku, Osaka, 532, Japan*

*A versatile automated apparatus, equipped with an artificial intelligence has been developed which may be used to prepare and isolate a wide variety of compounds. The prediction of the optimum reaction conditions and the reaction control in real time, are accomplished using novel kinetic equations and substituent effects in an artificial intelligence software which has already reported* [1]. *This paper deals with the design and construction of the fully automated system, and its application to the synthesis of a substituted N-(carboxyalkyl)amino acid. The apparatus is composed of units for perfoming various tasks, e.g. reagent supply, reaction, purification and separation, each linked to a control system. All synthetic processes including washing and drying of the apparatus after each synthetic run were automatically performed from the mixing of the reactants to the isolation of the products as powders with purities of greater than 98%. The automated apparatus has been able to run for 24 hours per day, and the average rate of synthesis of substituted N-(carboxyalkyl)amino acids has been three compounds daily. The apparatus is extremely valuable for synthesizing many derivatives of one particular compound structure. Even if the chemical yields are low under the optimum conditions, it is still possible to obtain a sufficient amount of the desired product by repetition of the reaction. Moreover it was possible to greatly reduce the manual involvement of the many syntheses which are a necessary part of pharmaceutical research.*

### Introduction

In pharmaceutical research it is often necessary to synthesize many derivatives of one particular active compound to seek out the relationship between the chemical structure and the biological activity. Although laboratory automation has significantly reduced the manual involvement in pharmaceutical research, with instruments for automated batch-type reactions becoming available [2–4], it has generally been aimed at automation of bench-scale reactions to determine the best synthetic route or optimum reaction conditions prior to pilot-scale work. The instruments are rarely equipped with automated systems for purification and isolation of the products and, furthermore, the reaction optimization often utilizes analytical techniques which require relativelly long time-scale measurement, e.g. high performance liquid chromatography (HPLC). This severely limits the applicability of the instrument for syntheses with rapid and/or multi-step reactions, especially those with unstable or undetectable intermediates.

A versatile automated apparatus has been developed which is equipped with an artificial intelligence. It may be used to prepare and isolate a wide variety of compounds. This paper deals with the design and construction of the fully automated system, and shows an application of it to the synthesis of a substituted N-(carboxyalkyl)amino acid. The novel kinetic equations and substituent effects which make up the artificial intelligence, have previously been reported [5] and the synthesis of over two hundred novel compounds will later be presented elsewhere[6].

### Design and construction of the hardware

The automated apparatus may be divided into two parts: the computer and synthesis systems. The general appearance and a block diagram of the apparatus are shown in figures 1, 2 and 3 respectively.

The computer system consists of two personal computers (NEC PC-9801 and Fujitsu FM-77AV), two printers, two colour CRT monitors and interfaces. The synthesis system consists of a series of units, which have the following functions: supplying reactants; performing reactions; purifying products; freeze-drying; heating/cooling; washing; and handling exhaust/waste.

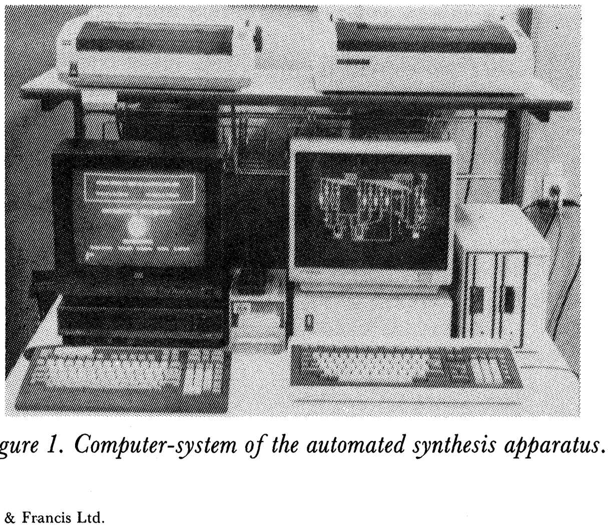

*Figure 1. Computer-system of the automated synthesis apparatus.*

0142–0453/89 $3.00 © 1989 Taylor & Francis Ltd.

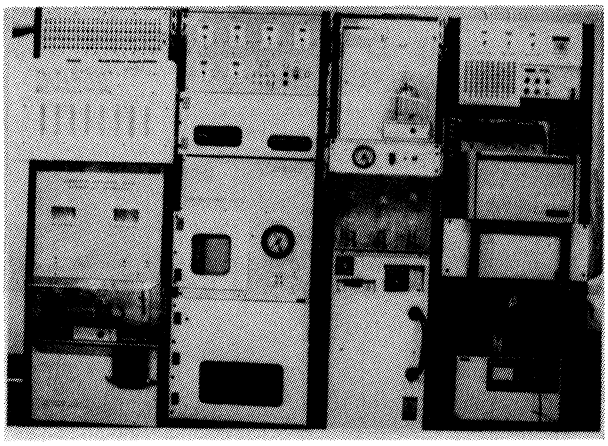

*Figure 2. Synthesis-system of the automated apparatus.*

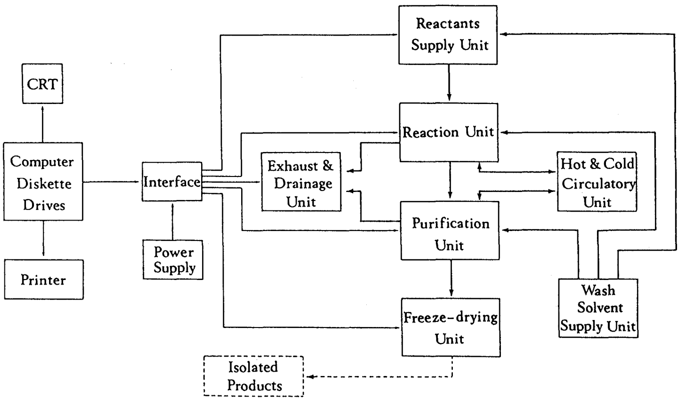

*Figure 3. Block diagram of the automatic apparatus.*

### *1. Reactant supply unit*

The reactant supply unit (figure 4) has two separate devices for supplying raw materials. The first device consists of seven reservoirs, nineteen solenoid valves and a volumetric tube (5 ml) equipped with a gas-liquid boundary sensor (G–L sensor). The second supply device has two reservoirs, ten solenoid valves and a volumetric tube (10 ml) fitted with a G–L sensor. All the flow lines are part of a closed system, thus lines WA 1, 2 and 3 in figure 4 are connected to the wash-solvent supply unit, and flow lines EX 1 and 2 are conneted to the exhaust/drainage unit. The transfer of liquid reactants is performed in two steps: firstly, the reactant is allowed to flow from the reservoir into the volumetric tube. Then, when the tube is full and the G–L sensor is activated, the contents of the volumetric tube are emptied into the reaction flask RF1. The same volume of a reactant solution may be repeatedly measured and added to the reaction flask by using the same sequence of control signals from the computer. After a synthetic run, all of the flow lines and reservoirs are washed and dried by passing wash solvents and then compressed air through them.

### *2. Reaction unit*

The reaction unit (figure 5) consists of six reagent supply devices, two reaction flasks, a separation funnel equipped with a liquid-liquid boundary sensor (L–L sensor), forty-

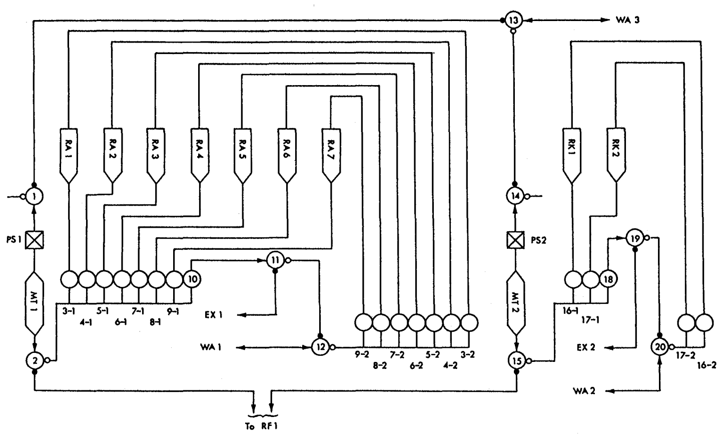

*Figure 4. Diagram of the reactant supply unit. RA = reservoirs of amino acid derivatives; RK = reservoirs of keto acids; PS = G–L sensors; MT = volumetric tube; WA = to wash unit; EX = to exhaust pump; RF = to reaction flask; ◯ = solenoid valves.*

seven solenoid valves and a pH adjusting flask fitted with a pH electrode.

The reaction flasks, RF1 and RF2, are both approximately 100 ml in volume and have jackets through which the heating/cooling fluid of 50% aqueous polyethylene glycol (Nisso Yuka KK, Japan) is circulated.

The reaction temperature is maintained at the desired setting by regulating the flow around the flask of either hot or cold fluid. The reagent supply devices are fundamentaly the same as those described for the reactants. The maximum dropping rate of the solutions from the volumetric tubes, MT3 and MT8, was 0·41 ml/sec (minimum rate, 0·085 ml/sec) using the computer control-signals. The reaction mixture is transferred under reduced pressure from flask RF1 to RF2 for further reaction or treatment, then to the separation funnel, and finally to the pH adjusting flask. Solvent extraction or washing of the product mixture may be performed in the separation funnel. The L–L sensor detects the boundary between two phases by the difference in their electrical resistance. Thus valve number 44 may be opened and shut to separate two phases when the L–L sensor is activated, and the required one is allowed to flow into RF3. The pH of the final solution is adjusted as necessary by titrating with acid or base, held in the MT8 volumetric tube, before it is automatically injected into the purification unit. All of the flow lines are washed and dried after each run.

### *3. Purification unit*

The purification unit consists of two devices: one using preparative HPLC (figure 6) and the other centrifugal partition chromatography, CPC (figure 7). The devices may be used separately or consecutively as required. The HPLC device consists of three columns, a pump, an UV-detector, a refractive index detector (RI), ten solenoid valves, two six-way rotary valves (Rheodyne, USA) fitted with G–L sensors, two transfer pumps and two four-way rotary valves. The CPC device includes a

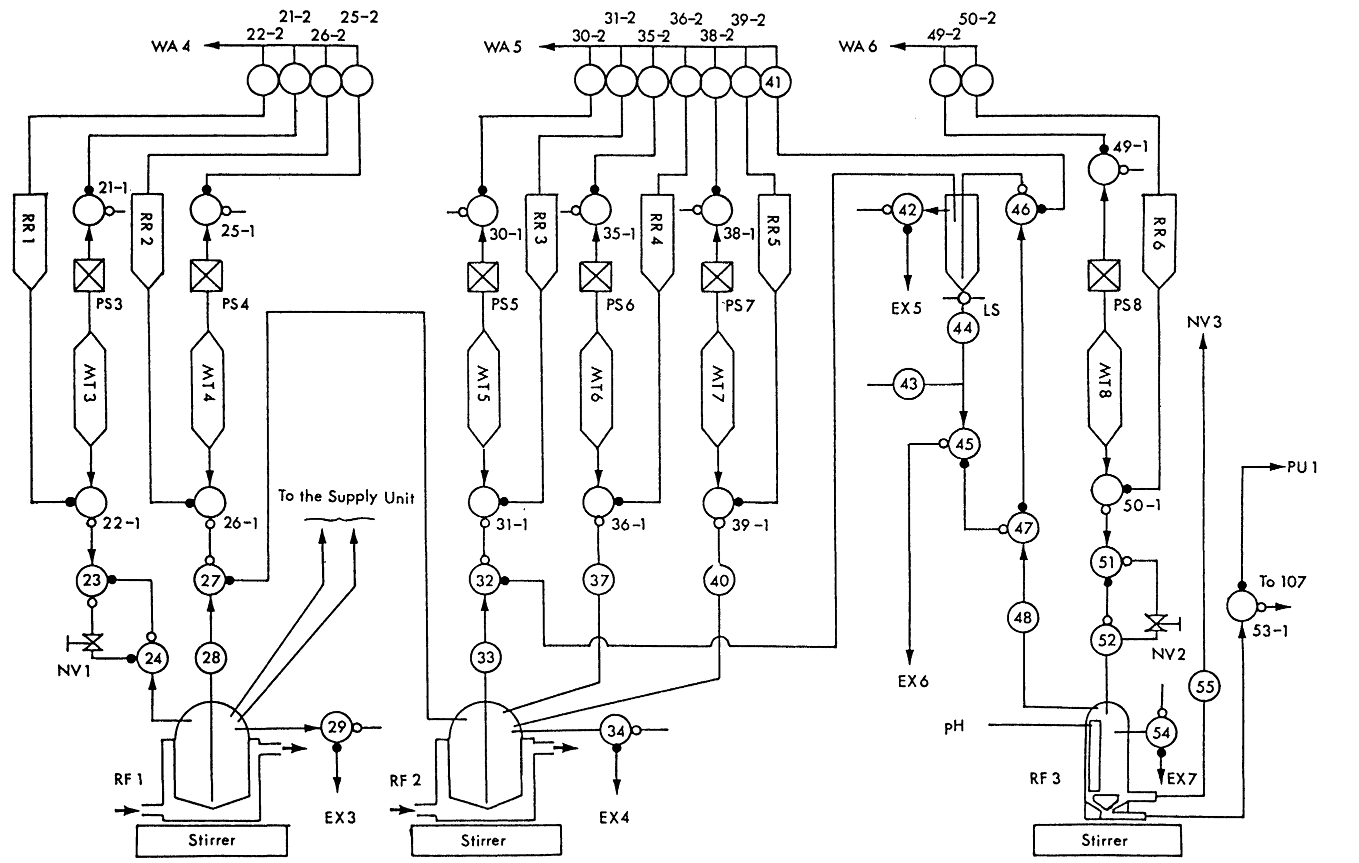

*Figure 5. Diagram of the reaction unit. RR = reagent reservoirs; PS = G–L sensors; RF = reaction flasks; NV = needle valves; LS = L–L sensor; pH = pH meter; MT = volumetric tubes; ○ = solenoid valves; → = from hot and cold circulatory unit.*

six-way rotary valve fitted with a G–L sensor, a four-way rotary valve, an UV detector, a transfer pump, thirty one solenoid valves and an evaporator. The evaporator is equipped with a solvent supply line, for dissolving solid products, and a sensor which detects the completion of evaporation by the temperature difference of the vapor stream. Automatic injection is performed by the transfer pumps connected to the Rheodyne valves fitted with G–L sensors. In order to avoid injection of air into the HPLC column, it is necessary to remove the residual air, in the flow line between the RF3 flask and 6-way Rheodyne injection valve. This is accomplished by passing the HPLC mobile phase through the flow line, from the valve to the flask, using the transfer pump. After the flow line is filled with the solution, the transfer pump is reversed and the whole of the sample in the RF3 flask is drawn into a sample loop fitted onto the six-way valve. When air is finally drawn up the flow line from the RF3 flask, the G–L sensor signals for the immediate rotation of the Rheodyne valve from the load to the injection position. The eluent from the column is monitored by UV or RI, and the peaks which differ in retention time from the starting materials are collected in frations. A chromatographic chart is displayed on the CRT monitor, stored on disk, and later printed.

All flow lines including volumetric tubes and flasks used during the course of the synthesis are washed with water and methanol, and then dried by compressed air. After the power switches of the detector and the recorder are turned off,the separation column is washed with 20% methanol stored in SR2 and an additional one hour with water stored in SR1.

*4. Additional service units*

The isolated product after purification may be transferred to a freeze-drying unit as show in figure 8. The washing and exhaust drainage unit, and the temperature control circulatory unit are shown in figures 9 and 10, respectively.

### Software for the automated synthesis

The automated synthesis apparatus is controlled by a PC-9801 and FM-77AV personal computers equipped with input and output interfaces. The automation software is written primarily in BASIC, and the flow charts of the program modules are shown in figures 11, 12 and 13.

As shown in figure 11, the user assembles information (Input-A) concerning reaction performance through dialogue with an interactive program before the beginning of the reaction operation. The input information is stored in a random access memory, and then all subsequent experimetal operations proceed automatically without dialogue. The selection of amendment (Choice-B) means that an unnecessary operation step can be omitted by the Process-A. Since physical constants are

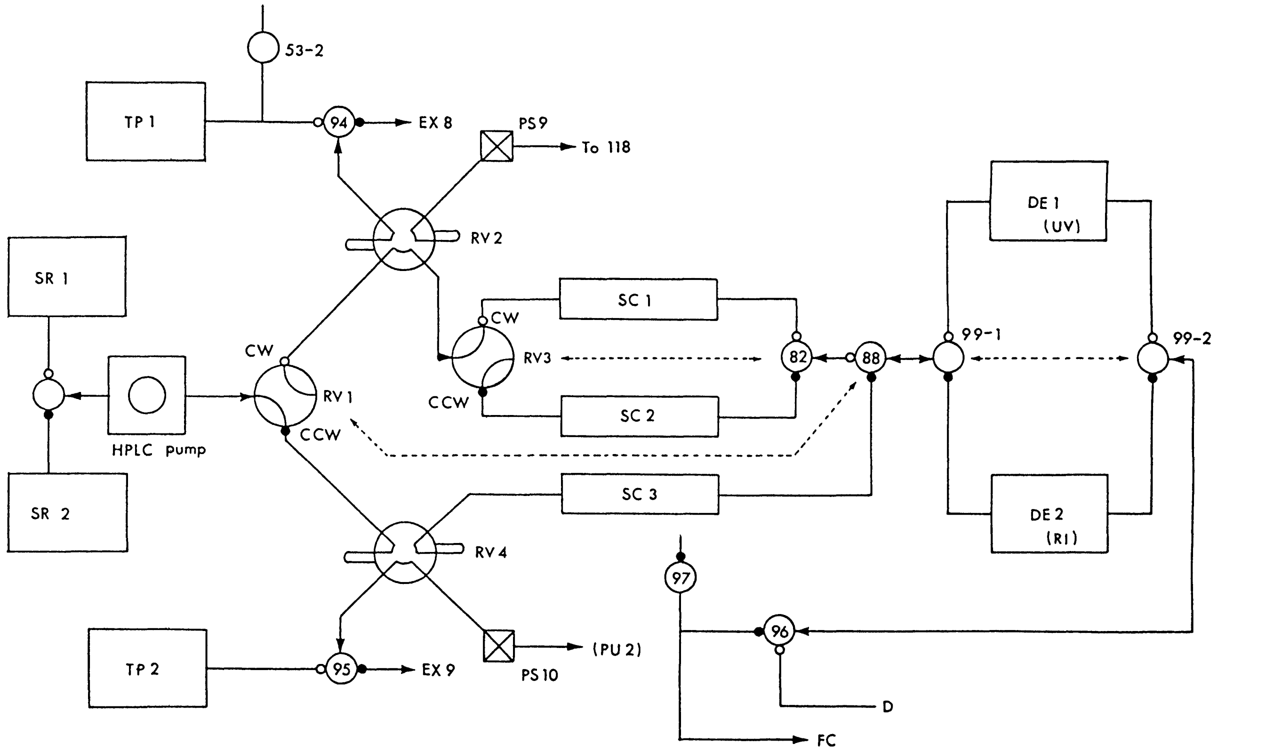

*Figure 6. Diagram of the purification unit: HPLC device. TP = transfer pump; SR = solvent reservoirs; RV = rotary valves (6-way and 4-way valves); PS = G–L sensor; DE = detectors; EX = to exhaust unit; CW = clockwise; CCW = counterclockwise; SC = HPLC column; FC = fraction collector; D = to CPC device; ○ = solenoid valves.*

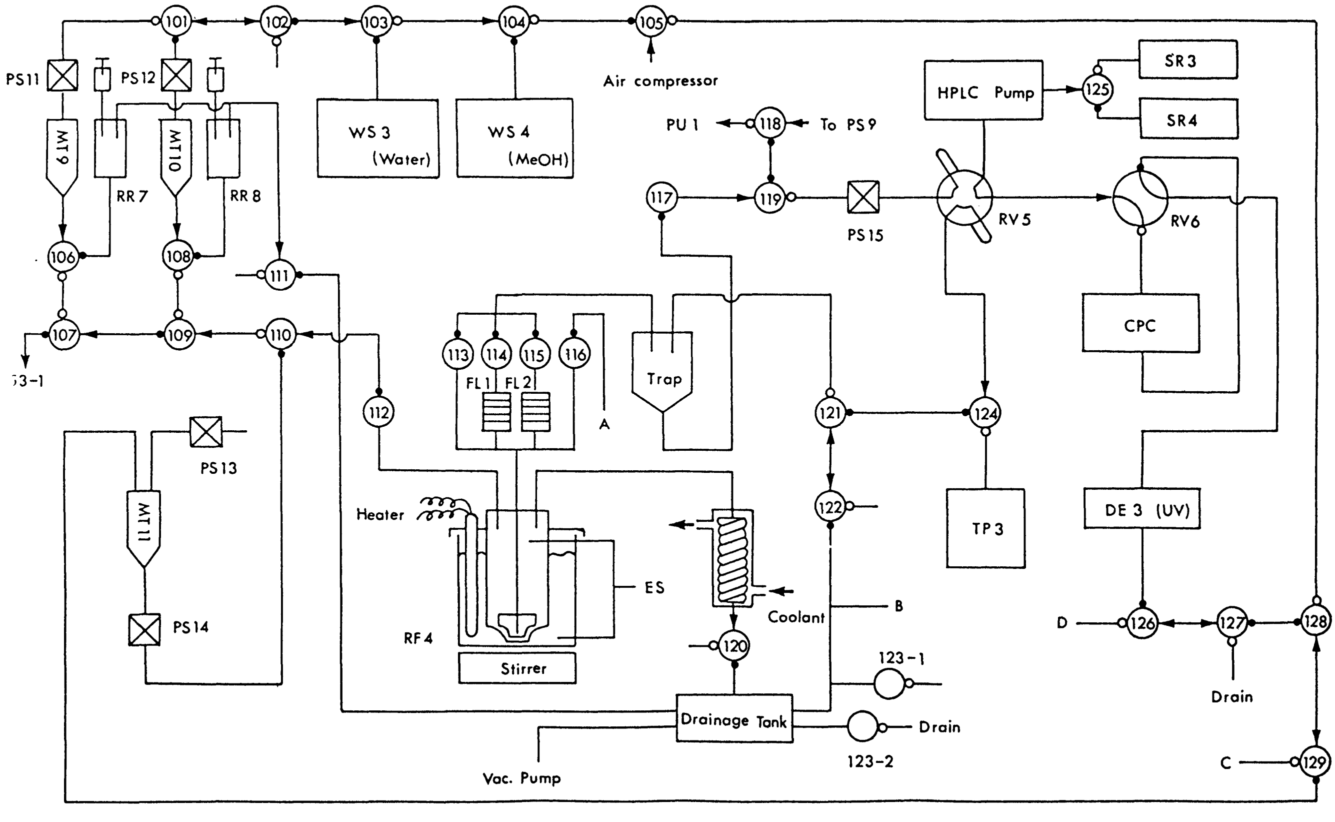

*Figure 7. Diagram of the purification unit: CPC device. PS = G–L sensor; WS = washing solvents; RF = evaporation flask; FL = filters; RV = rotary valves; SR = solvent reservoirs; ES = evaporation sensor; DE = detector; RR = reagent reservoirs; MT = volumetric tube; A,B and C = to freeze-drying unit; D = from HPLC device; ○ = solenoid valves.*

maintained on a magnetic disk, in the case of auto mode, the kinetic constants used for the prediction of the optimum reaction conditions are called by referring to the reaction name and the subsituent name of the raw material (figure 12).

If no kinetic data is found in the data file, new input information is requested and then stored on the magnetic disk. After some rate constants or analytical kinetics data of a reaction series are accumulated, it is possible to calculate the reaction constant or substituent constants

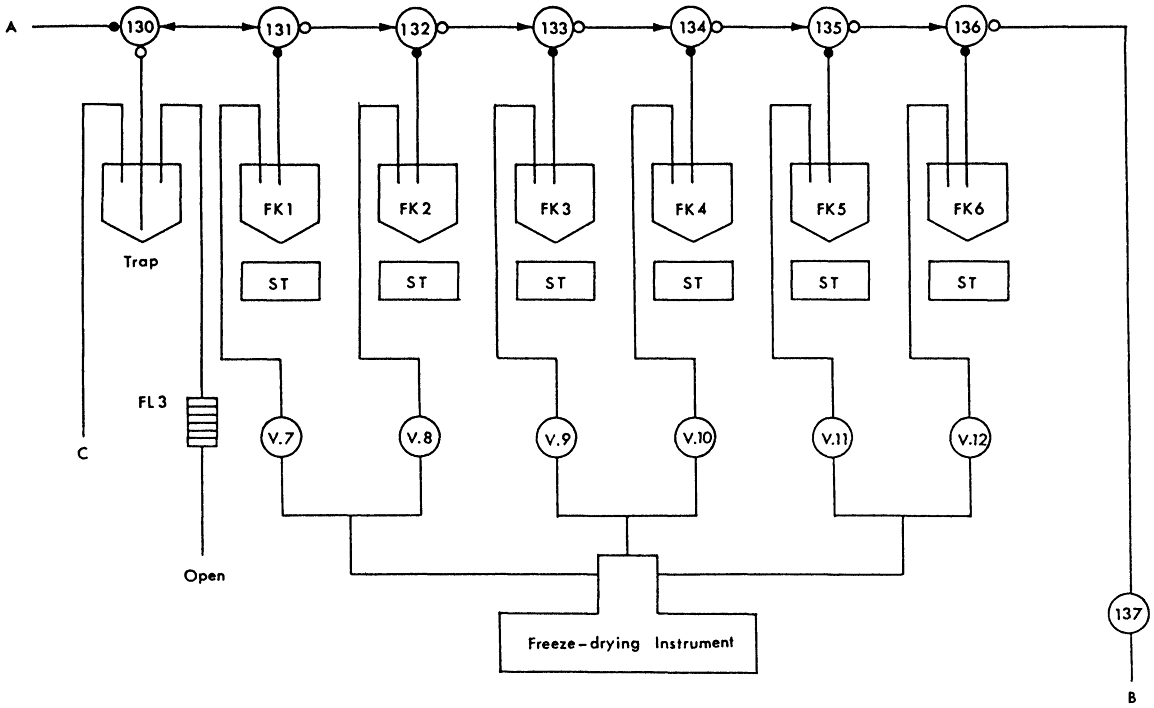

*Figure 8. Diagram of the freeze-drying unit. ST = magnetic stirres; A, B and C = lines from purification unit; FK = freeze-drying flasks; FL = filter; ○ = valves.*

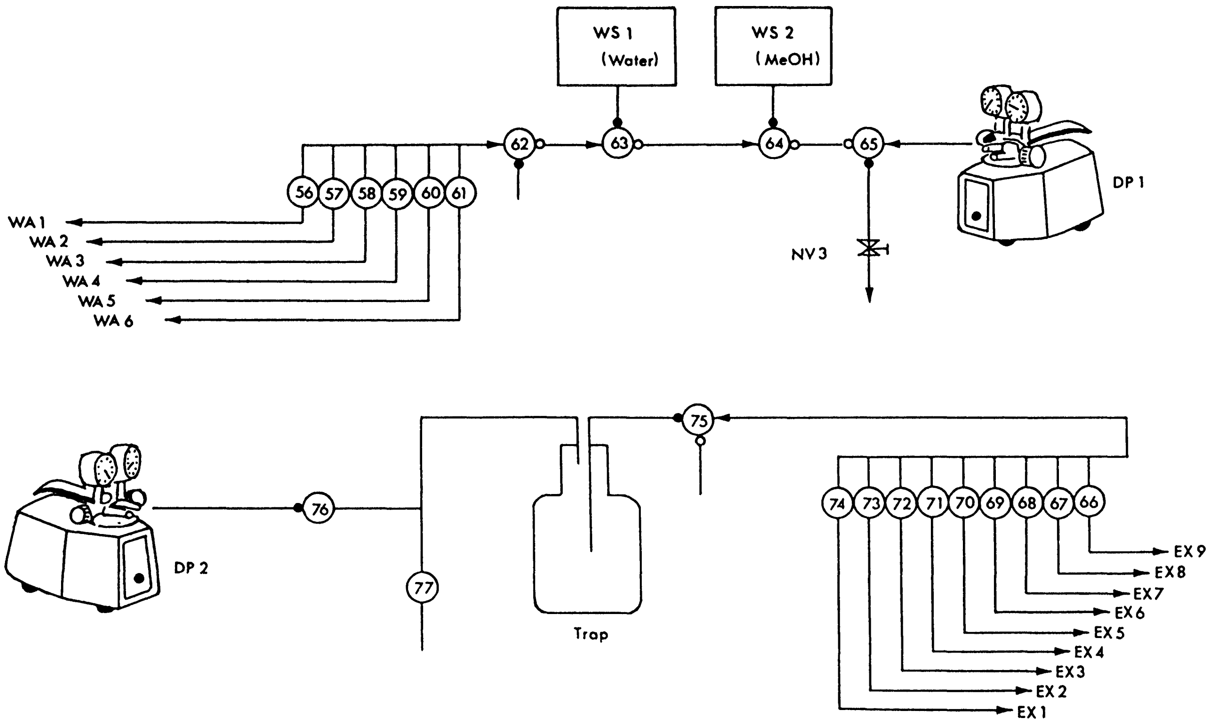

*Figure 9. Diagram of the washing and exhaust/drainage unit. WS = washing solvents; NV = needle valve; DP = diaphram pumps; ○ = valves.*

by using the calculation subroutines (Calculation-A and B). Optimum reaction conditions are computed by the Process-B and used for controlling the reaction. The experimental operations are presented in figure 13. The initialization gets the apparatus ready for a reaction; the syringe pumps in the purification unit are reset to their top-of travel positions and the six-way valve of the HPLC set to the sampling position. The Process-C executes distribution of raw materials, reagents and solvents to reaction flasks and volumetric tubes, according to the combination and sequence of reactions decided in the input information. The Process-D executes reactions, concentration of a reaction mixture, transfer between flasks for the next reaction step or treatment, and extraction. If necessary, pH of the reaction mixture may be adjusted by titrating it with an alkali or acid solution, before injection to the HPLC or CPC column. The Process-E executes measurement of HPLC retention-time, purity of separated product and yield, if the molar absorption coefficient is available on the data disk. The purity and chromatographic chart are stored in a record file and printed out later. If the retention times of HPLC are identical to those of the raw materials, the eluate of the raw material is passed to the exhaust/drainage unit.

The product fraction isolated is concentrated to dryness under reduced pressure or freeze-dried (Process-G). When the operation program has terminated, the Process-F executes washing of the flasks, valves and flow lines to get ready for a new synthesis, if so directed in the input information. If the product yield is lower than the required value, the operation sequence continues to repeat until the appropriate termination condition is met (Decision-B).

### Automated synthesis of a substituted *N*-(carboxyalkyl)amino acid

The functions of the automated synthesis apparatus were confirmed by performing a typical reaction. The reaction of *L*-isoleucine tert-butyl ester acetic acid salt (I) with 2-ketocaproic acid sodium salt (II) in methanol gives the unstable intermediate, Schiff base, which is reduced with sodium cyanoborohydride to afford *N*-(1-sodiocarboxy-*n*-pentyl)-*L*-isoleucine tert-butyl ester (III). The equilibrium and the consecutive reactions are controlled by adding sodium cayanoborahydride using the artifical intelligence software, which contains novel kinetic equations and substituent effects[5].

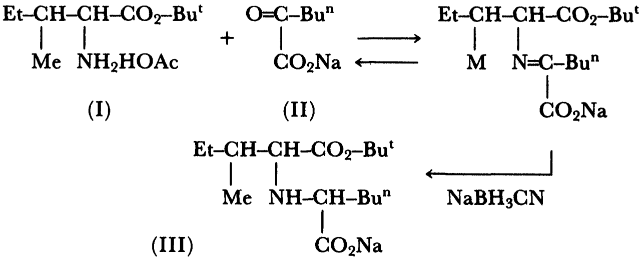

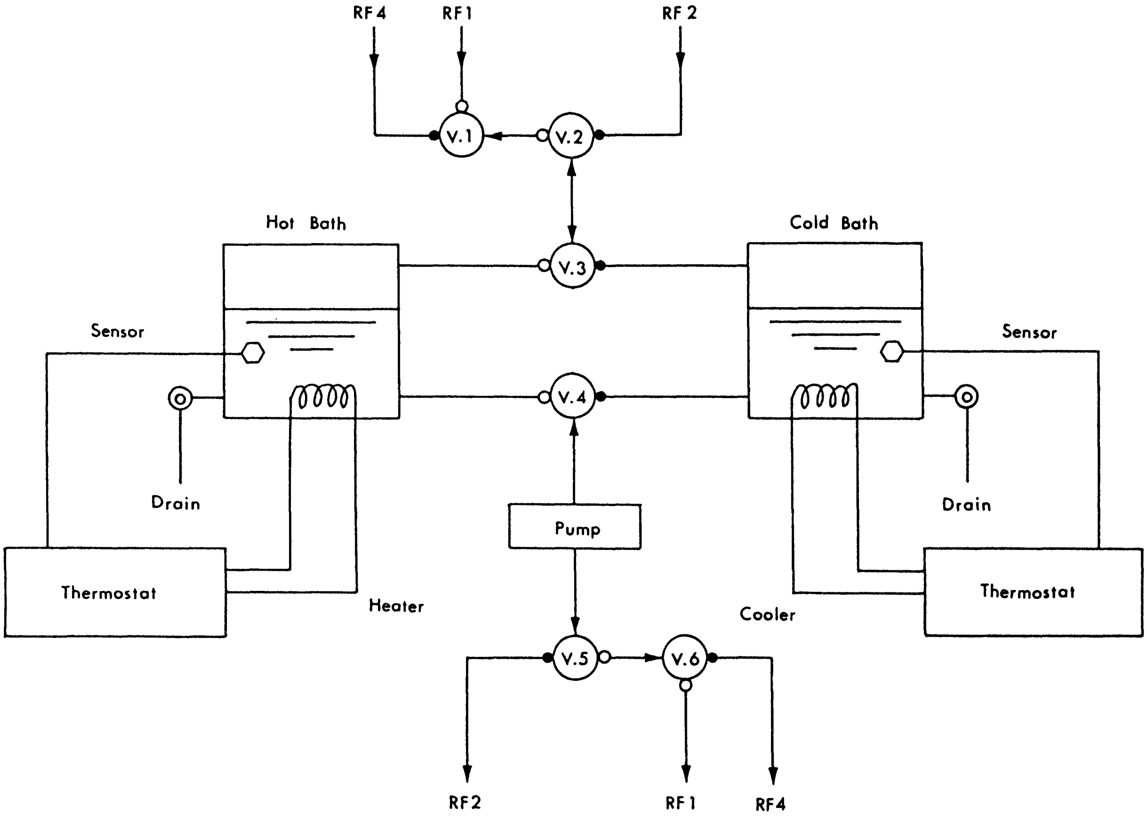

*Figure 10. Diagram of the temperature control circulatory unit. RF = to reaction flask; ○ = valves.*

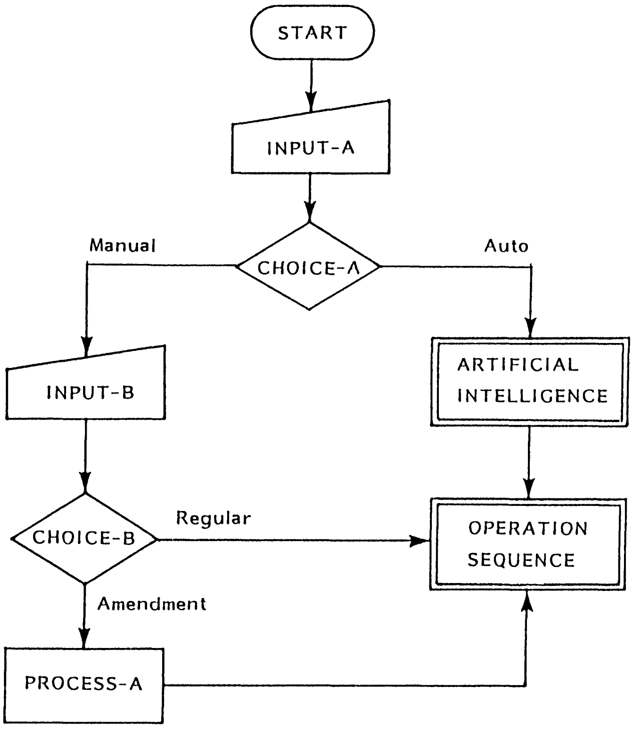

*Figure 11. Program module flowchart. Input-A: (1) Reaction and product name; (2) Names of raw materials, reagents and solvents (reservoir number and volume to be use); (3) Purification conditions (stationary and mobile phases, retention time of the raw material, pH-adjustment to be specified). Input-B: Reaction conditions (reaction temperature and time, repetition). Process-A: Amendment of operational sequence.*

Methanolic solutions of I (0·8 mol/l), II (0·4 mol/l), sodium cyanoborohydride (0·2 mol/l) and aqueous sodium hydroxide (0·8 mol/l; MeOH:$H_2O$=1:1, v/v) were stored in the reservoirs RA1, RK1 and RK4, respectively. Under computer control, 5 ml of I and 10 ml of II were volumetrically measured and added to the RF1 reaction flask. The rate of addition of sodium cyanoborhydride solution was controlled as shown in table 1. The artificial intelligence directs the addition of sodium cyanoborohydride until the rate of decrease of the chemical yield against reaction time reaches 0·1% per minute. The reaction is terminated 10 minutes after the final addition.

Thus the reaction was stopped after 103 minutes, and the resulting mixture was transferred from RF1 to RF2 flask. After evaporation to dryness under reduced pressure, 10 ml of aqueous sodium hydroxide was added to the residue and then it was injected into the purification unit. The chromatographic chart which was displayed on the CRT monitor during the purifiction stage, is presented in figure 14.

The peak at retention time 29·5 min was not clearly identified but it was attributed to a decomposition product of the sodium cyanoborohydride and the keto acid. The two peaks at 61·5 and 83·3 min were identical to those of the diastereoisomers of III. The fractions collected at these two peaks were transferred to the freeze-drying unit. The analytical yield of these two peaks was 88·3% on the basis of the intensities of the chromatographic chart. However a fraction having the retention

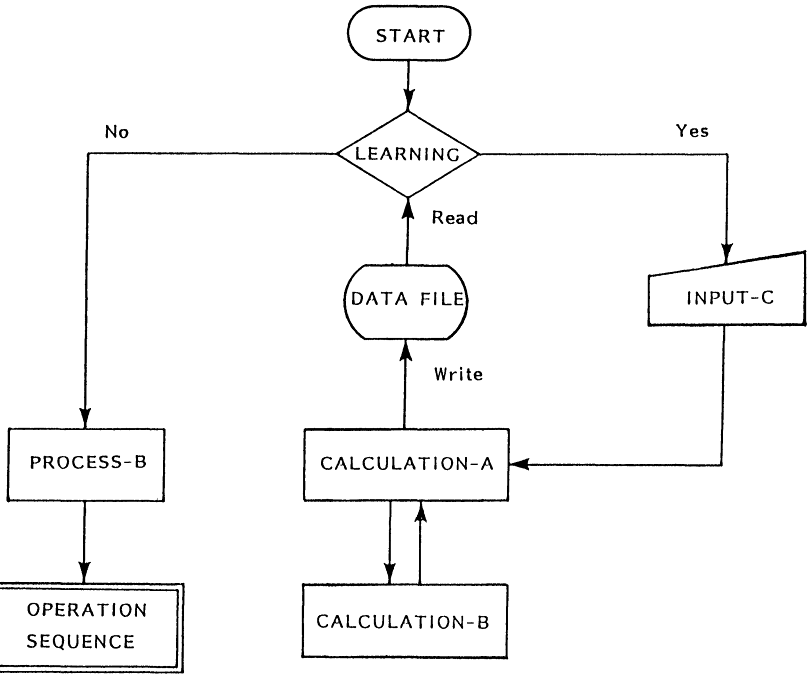

*Figure 12. Flowchart of the artificial intelligence. Input-C: Analytical kinetics-data of the reaction. Process-B: Calculation of the optimum reaction conditions. Calculation-A: calculation of reaction and substituent constants. Calculation-B: Calculation by the least squares method.*

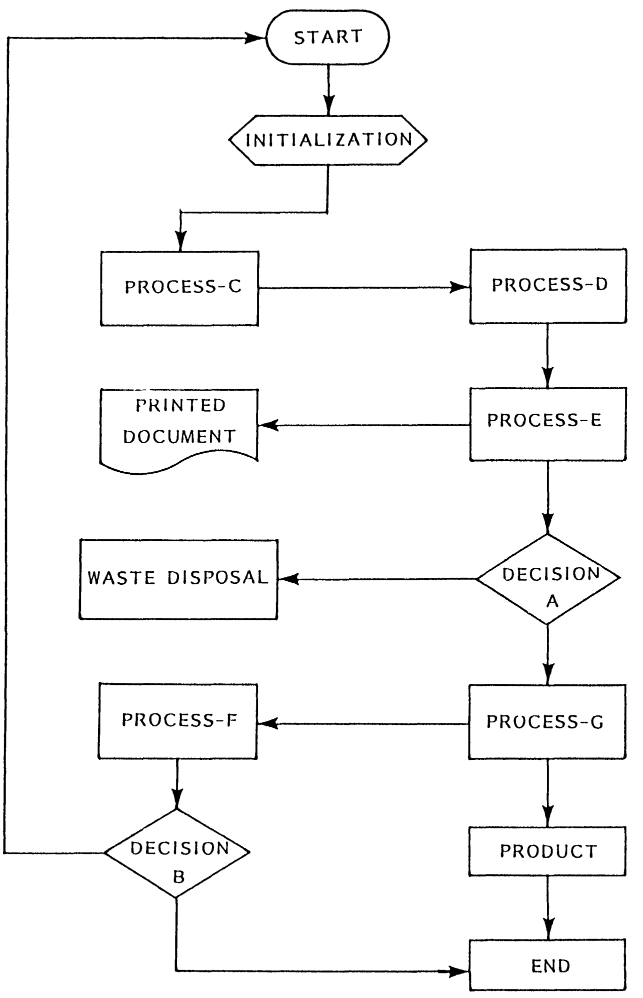

*Figure 13. Flowchart of the operation sequence. Process-C: Supply of raw materials, reagents and solvents (volumetric measurement and distribution to flasks). Process-D: Reaction control and treatment (reaction time and temperature, concentration, extraction, and transfer between flasks). Process-E: Purification (measurement of retention time, purity and yield). Process-F; Washing (flasks, valves and flow lines). Process-G: Isolation and concentration of product (freeze-dry).*

times between 66·8 and 73·7 min was not collected. Therefore the combined synthetic yield of both isomers of III (III-a and III-b) was 78·2%.

### Conclusion

The automated apparatus was able to run for 24 hours per day, and the average rate of synthesis of *N*-(carboxyalkyl)-amino acids had been three compounds daily. The apparatus is extremely valuable for synthesizing many derivatives of one particular compound structure. Even if the chemical yields are low under the optimum conditions, it is still possible to obtain a sufficient amount of the desired product by repetition of the reaction. Moreover, it is possible to greatly reduce the manual movement of the many syntheses which are a necessary part of pharmaceutical research.

*Table 1. Addition control table of sodium cyanoborohydride*

| Addition time (min) | Addition volume of cyanoborohydride (ml) |
|---|---|
| 2·8 | 4·5 |
| 8·1 | 3·0 |
| 17·4 | 2·0 |
| 32·5 | 1·3 |
| 56·3 | 0·9 |
| 93·1 | 0·6 |
| 103·1 | Finish |

### Experimental

*Purification and identification of products*

The purification of products in the automated synthesis was carried out using a preparative column chromatograph, which was operated as follows: column XAD-2 (50×2 cm); detector: UV-235 nm; mobile phase, $H_2O$:MeOH=1:1 (v/v); flow rate: 6 ml/min.

The diastereoisomers, III-a and III-b, were further purified and analyzed.

*III-a.*

Analysis calculated for $C_{16}H_{30}NO_4Na$ 0·5$H_2O$: C, 57·81; H, 9·40; N, 4·21.

Found: C, 58·01; H, 9·30; N, 4·18. IR $\nu_{max.}^{KBr}$ ($cm^{-1}$): 3420, 2960, 2930, 2870, 1722,1580, 1419, 1365, 1155. UV nm (ε): 245 (47), 235 (140), 225 (307). $[\alpha]_D$: −19·9° (c=1·14 in $H_2O$). $^1H$ NMR (ppm in $D_2O$) δ: 0·884 (*Me*$CH_2$–, 3H, t,J=7·1), 0·898 (*Me*$CH_2CH_2$–, 3H, t,J=7·2), 0·906 (*Me*CH–, 3H, d,J=6·8), 1·744 (MeC*H*–, 1H, m), 1·20–1·37 and ca. 1·5 (C$H_2$–, 6H, m), 1·498 (tert-Bu, 9H, s), ca. 1·57 (MeC$H_2$CH–, 2H, m), 2·944 (C*H*-$CO_2$Na, 1H, dd,J=5·9 and 7·6), 3·007 (C*H*-$CO_2$-tert-Bu, 1H, d,J=6·6).

*III-b.*

Analysis calculated for $C_{16}H_{30}NO_4Na$: C, 59·42: H, 9·35; N, 4·33.

Found: C, 59·08; H, 9·07; N, 4·29. IR $\nu_{max.}^{KBr}$ ($cm^{-1}$): 3420, 2955, 2930, 2865, 1715, 1580, 1415, 1365, 1160. UV nm (ε): 245 (98), 235 (201), 225 (333). $[\alpha]_D$: −6·2 (c = 0·945 in $H_2O$). $^1H$ NMR (ppm in $D_2O$) δ: 0·889 (*Me*$CH_2$–, 3H, t, J = 7·1), 0·920 (*Me*$CH_2CH_2$–, 3H, t, J = 7·2), 0·911 (*Me*CH–, 3H, d, J = 7·1), 1·46 and 1·20–1.35 (C$H_2$–, 6H, m), 1·477 (tert-Bu, 9H,s), 1·584 (NHCH-C$H_2$–, 2H, m), 1·727 (MeC*H*–, 1H, m), 2·986 (C*H*-$CO_2$Na, 1H, t, J = 6·3), 3·156 (C*H*-$CO_2$-tert-Bu, 1H, d, J = 5·1).

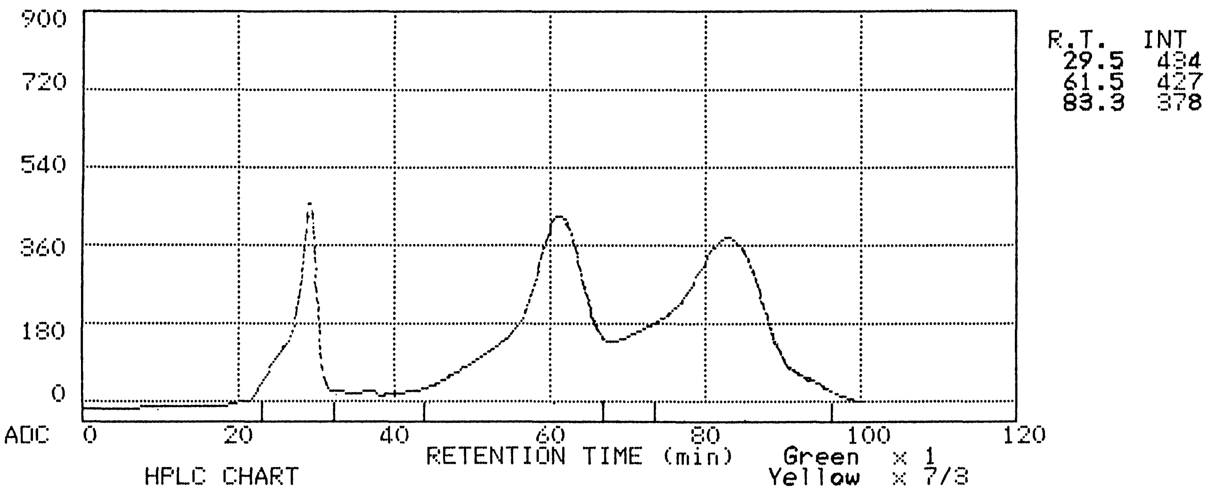

*Figure 14. HPLC chart of the reaction mixture after purification.*

### References

1. Hayashi, N. and Sugawara, T., *Chemistry Letters (Japan)*, (1988), 1613.
2. Legrand, M. and Bolla, P., *Journal of Automatic Chemistry*, **7** (1985), 31.
3. Winicov, H., Schainbaum, J., Buckley, J., Longino, G., Hill, J., and Berkoff, C. E., *Analytical Chimica Acta*, **103** (1978), 469.
4. Frisbee, A. R., Nantz, M. H., Kramer, G. E. and Fuchs, P. L., *Journal of the American Chemical Society*, **106** (1984), 7143.
5. Hayashi, N. and Sugawara, T., *Tetra. Computer Methodology*, **1** (3) (1989), in press.
6. Hayashi, N., Sugawara, T. and Kato, S., in preparation.

